# Perioperative changes in haemoglobin and ferritin concentrations from preoperative intravenous iron isomaltoside for iron deficiency anaemia in patients with colorectal cancer: A pilot randomised controlled trial

**DOI:** 10.1371/journal.pone.0270640

**Published:** 2022-06-30

**Authors:** Pui Lam Polly Fung, Vivian Nga Man Lau, Floria Fung Ng, Wing Wa Leung, Tony Wing Chung Mak, Anna Lee

**Affiliations:** 1 Department of Anaesthesia and Intensive Care, Prince of Wales Hospital, Shatin, New Territories, Hong Kong SAR, China; 2 Department of Anaesthesia and Intensive Care, The Chinese University of Hong Kong, Shatin, New Territories, Hong Kong SAR, China; 3 Division of Colorectal Surgery, Department of Surgery, The Chinese University of Hong Kong, Shatin, New Territories, Hong Kong SAR, China; Shahid Beheshti University of Medical Sciences, ISLAMIC REPUBLIC OF IRAN

## Abstract

**Background:**

Patients with colorectal cancer have a high risk of iron deficiency anaemia (IDA) due to chronic tumour induced blood loss, a reduced dietary iron intake from poor nutrition or gastrointestinal malabsorption. This pilot, double blinded, randomised controlled trial (RCT) examined the effect and feasibility of using preoperative iron isomaltoside for treating iron deficiency anaemia.

**Methods:**

Forty eligible adults with IDA were randomised to receive either intravenous iron isomaltoside (20 mg.kg^-1^ up to 1000 mg over 30 minutes) or usual preoperative care (control) three weeks before scheduled colorectal surgery. The primary outcomes were perioperative changes in haemoglobin and ferritin concentrations.

**Results:**

The recruitment rate was 78% of all eligible referred patients (1.9 patients/month). The haemoglobin and ferritin concentrations were higher in the iron isomaltoside group than the control group over the perioperative period (group*time interaction *P* = 0.042 and *P* < 0.001 respectively). Mean haemoglobin change from baseline to before surgery was higher in the iron isomaltoside group (7.8, 95% CI: 3.2 to 12.3 g.l^-1^) than the control group (1.7, 95% CI: -1.9 to 5.3 g.l^-1^) [mean difference 6.1, 95% CI: 0.3 to 11.8 g.l^-1^; *P* = 0.040]. The ferritin change from baseline to before surgery between groups was large in favour of the iron isomaltoside group (mean difference 296.9, 95% CI: 200.6 to 393.2 μg.l^-1^; *P* < 0.001]. There were no differences between groups in packed red blood cell transfusions needed, surgical complications, quality of recovery and days (alive and) at home within 30 days after surgery.

**Conclusion:**

Iron isomaltoside therapy was safe and had a minimal effect on perioperative changes in haemoglobin concentration. Given the slow recruitment and new evidence emerging during the conduct of this study, conducting a multi-centre RCT based on the current pilot trial protocol is unlikely to be feasible.

**Trial registration:**

ClinicalTrials.gov NCT03565354.

## Introduction

The global burden of colorectal cancer is substantial. It is the third most commonly diagnosed cancer (10.2% of total new cases) and the second leading cause of cancer-related mortality (9.2%) worldwide [1]. Patients with colorectal cancer have a high risk of iron deficiency anaemia (IDA) due to chronic tumour induced blood loss, a reduced dietary iron intake from poor nutrition or gastrointestinal malabsorption [2]. In a cohort study of the 735 patients undergoing colorectal cancer resection, 427 (58%) had anaemia (haemoglobin less than 130 g.l^-1^), with absolute iron deficiency being the most common (approximately 80%) cause of anaemia [3].

While preoperative anaemia is one of the strongest predisposing risk factor for perioperative allogeneic blood transfusion [4], allogeneic blood transfusion itself is associated with higher risks of all-cause mortality, postoperative infections, surgical re-intervention [5] and cancer recurrence [6]. Thus, timely correction of preoperative iron deficiency, as part of patient blood management, by iron therapy is an alternative to allogeneic blood transfusion.

This study uses iron isomaltoside 1000 (Monofer®), an unbranched oligosaccharide where iron is tightly bound within a matrix structure, which has potentially less oxidative stress and immunological toxicity compared to other parenteral iron formulations [7]. This property allows iron isomaltoside infusion to be given at as a maximum single dose of 20 mg.kg^-1^ body weight (500 mg to 1500 mg/week), a higher weekly dose compared to ferric carboxymaltose (Ferinject®) at 20 mg.kg^-1^ body weight (500 mg to 1000 mg/week) [8]. While there are a few published randomised controlled trials (RCTs) on the effect of preoperative intravenous iron therapy in patients with IDA scheduled for colorectal surgery, none have been conducted with iron isomaltoside [9–11]. The IVICA trial (n = 116) reported no difference in transfusion rate despite increase in haemoglobin with less anaemic patients at time of surgery after intravenous ferric carboxymaltose versus oral ferrous sulphate [11]. There were no patient-centred outcomes reported in the initial publication [11].

We conducted a pragmatic, single-centre pilot RCT to determine whether intravenous iron isomaltoside versus usual care (no iron supplement) in patients with IDA scheduled for colorectal resection was feasible. The objective was to examine the effect of a single intravenous iron isomaltoside administration for iron deficiency anaemia treatment on perioperative changes in haemoglobin and ferritin concentrations, and on patient-centred outcomes, with feasibility as a secondary outcome for conducting a larger multi-centre RCT.

## Methods

After approval from the Joint Chinese University of Hong Kong-New Territories East Cluster Clinical Research Ethics Committee (CREC Ref. No: 2018.128-T) and trial registration at ClinicalTrials.gov (NCT03565354), we performed a two-armed, parallel-group, 1:1 allocation ratio, double blinded, pilot RCT at the Prince of Wales Hospital, Hong Kong. All patients participating in the trial gave written informed consent. We followed the Consolidated Standards of Reporting Trials (CONSORT) guidelines for reporting pilot and feasibility trials in this paper [12, S1 Checklist].

We screened consecutive patients diagnosed with colorectal cancer listed for elective curative tumour resection operation from August 2018 to May 2020 for eligibility. Baseline blood tests including complete blood count, renal and liver function, iron profile (ferritin, transferrin saturation), C-reactive protein, vitamin B12 and folate were collected during the preoperative assessment. Patients were included if they were adults with anaemia (haemoglobin concentration < 130 g.l^-1^) and iron deficiency (ferritin < 30 μg.l^-1^ or 30 to 100 μg.l^-1^ with transferrin saturation < 20%) [8, 13].

The exclusion criteria included pregnancy or lactation; anaemia due to untreated vitamin B12 or folate deficiency, haemolytic disease, haemoglobinopathy, thalassemia or chronic renal failure on dialysis; iron overload (ferritin > 3000 μg.l^-1^ or transferrin saturation > 50%); haemochromatosis; previous ongoing iron replacement therapy or erythropoietin within 12 weeks before recruitment; known hypersensitivity towards iron isomaltoside; severe liver function derangement (aspartate aminotransferase or alkaline phosphatase exceeding three time upper limit of normal range); preoperative immunosuppressant therapy; or if surgery was scheduled less than three weeks from the screening date.

Patients were randomised to receive either intravenous iron isomaltoside (20 mg.kg^-1^ up to 1000 mg infused over 30 minutes) or usual preoperative care (no treatment) three weeks before scheduled colorectal surgery. The patient’s vital signs were charted before iron isomaltoside infusion, 15 minutes after the start, and immediately after the end of the infusion. We re-measured the haemoglobin concentration at two weeks after iron therapy intervention. A further intravenous dose of iron isomaltoside (same dose as the first) was given to the patient if the haemoglobin was less than 100 g.l^-1^.

The sequence generation was from a computer-generated random number list (no blocking) drawn by an investigator not involved in patient care. Treatment allocation was concealed in consecutive sealed opaque envelopes and handed over to a third party for arranging patients to receive preoperative intravenous iron therapy at an ambulatory surgical unit. No third-party contact was required if the patients were allocated to the control group. Patients were not blinded to the treatment allocation and were instructed not to take other forms of iron supplements during the study period. The surgical team and attending anaesthetists who were responsible for the patient’s perioperative management, including blood transfusions, were blinded to the treatment allocation. A research nurse, who collected the outcome data, was also blinded to the treatment allocation.

All participants attended the perioperative medicine clinic where standard preanaesthetic assessments took place. The clinical management of patients in both groups followed the local guidelines for colorectal cancer resection. Patients were admitted to the hospital one day before surgery as per usual clinical practice. The surgical team prescribed bowel preparation when necessary. There was no restriction in the choice of anaesthetic agents and analgesic modalities used on patients. We did not have a standardised trigger for blood transfusion, and it was left to the discretion of the attending anaesthetist and surgeon. Patient characteristics (age, sex, weight, height, diagnosis, ASA grade, major comorbidities), type of surgery, duration of surgery and amount of blood loss during surgery were extracted from the patient’s medical records.

The primary outcome were perioperative changes in haemoglobin and ferritin concentrations, especially between the interval from baseline (diagnosis date) to before surgery. The secondary outcomes were the number of units of packed red blood cells transfused during the perioperative period (randomisation date to hospital discharge); surgical complications occurrence and its severity was graded according to the Clavien-Dindo Classification [14]; duration of postoperative length of stay; quality of recovery (QoR-15) using the Chinese version 15-item questionnaire [15] on the third to fourth day after surgery and days (alive and) at home within 30 days after surgery (DAH30) [16]. The QoR-15 score ranged from 0 to 150, with items measuring pain, physical comfort, physical independence, psychological support and emotional state [15]. The validity (convergent, construct, discriminant), reliability (internal consistency, split-half, test-retest), responsiveness, acceptability and feasibility properties have been well established [15]. A poor symptom state (recovery) after surgery has been defined at a cut-off of less than 118 [17]. The DAH30 is a generic composite outcome incorporating the duration of postoperative hospital length of stay, discharge deposition (to home, rehabilitation centre or nursing home), hospital readmission and postoperative mortality [16]. Half a day difference is considered clinically meaningful [16]. Both QoR-15 and DAH30 are considered to be patient-centred outcomes [18]. A larger multi-centre trial was deemed viable if recruitment rate was more than 60% with 5 patients recruited each month and data completeness of laboratory and outcome variables was more than 90%.

Using the guidelines from Whitehead and colleagues [19], 20 (treatment) and 20 (usual care) achieved an 80% power to reject the null hypothesis of zero small effect size (0.20) for a change in haemoglobin concentration from baseline to pre-surgery using a two-sided two-sample equal-variance test at a significance level of 0.05. The estimated total sample size was 40.

We used complete case analysis. The normality of the data was assessed visually and by Shapiro-Wilk’s test. Parametric data were presented as mean and standard deviation (SD); between group comparisons were examined using the Student’s t-tests. Expected skewed data such as QoR score, DAH30 and number of units of blood transfusion were reported as median and interquartile range (IQR); between group comparisons were assessed using the Mann-Whitney U tests. We used the Chi-square test to compared categorical data between groups. A generalised estimating equation (GEE) model with a Gaussian distribution, identity link function, exchangeable correlation, and robust variance was used to estimate the main effect of treatment, time and group*time interaction for changes in haemoglobin, ferritin and transferrin concentrations across the four time points (baseline, before surgery, postoperative Day 1 and at hospital discharge). We analysed all of the data by the intention-to-treat method, with a 2-sided statistical significance level set at *P* < 0.05. Stata 16.1 (StataCorp, College Station, TX, USA) and SPSS 26.0 (IBM Corp, Armonk, NY, USA) software were used for data analyses.

## Results

**Fig 1:**
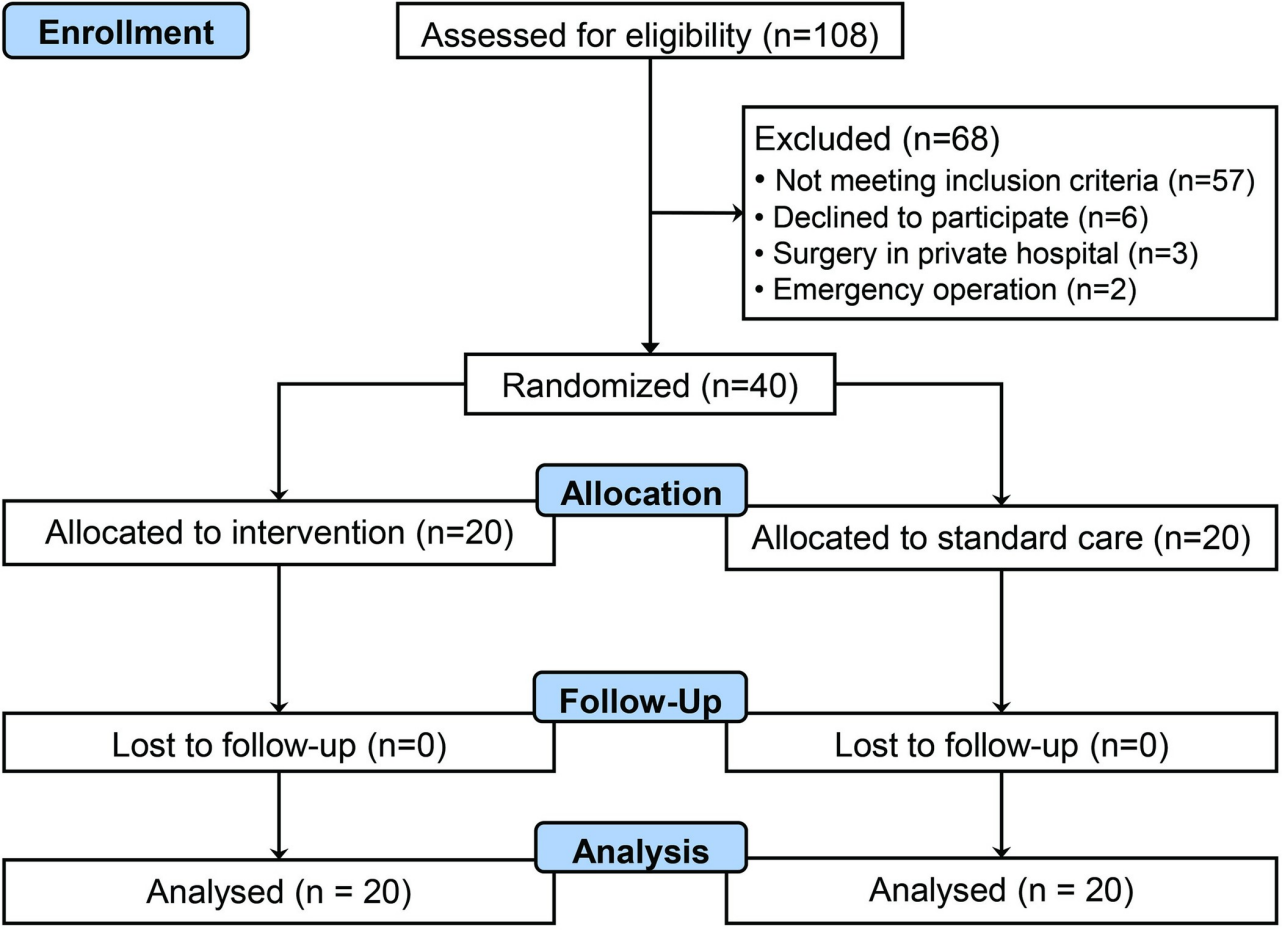
CONSORT diagram of patient recruitment.

During the study period from August 2018 to May 2020, 414 patients had undergone elective colorectal cancer surgery. Among those patients, 108 patients had a predicted waiting time to surgery of at least 3 weeks, and were screened for inclusion into the trial. Fifty-seven patients did not meet the inclusion criteria (48 did not meet the IDA criteria, 9 had haemoglobin concentration above 13 g.l^-1^). Among the 51 eligible patients, 6 declined to participate and 3 had surgery performed elsewhere and 2 were emergency cases (Fig 1).

Forty patients were randomised to receive either intravenous iron isomaltoside infusion (treatment group, n = 20) or usual care (control group, n = 20) (Fig 1). The recruitment rate was 78% (95% CI: 65% to 88%) of all eligible referred patients, at less than 2 patients per month. Laboratory and outcome data completeness was high (96.9%); all outcome measurements were complete but there were missing baseline C-reactive protein concentration (n = 4), postoperative day 1 ferritin concentration (n = 1), postoperative day 1 transferrin saturation (n = 1), discharge ferritin concentration (n = 2) and discharge transferrin saturation (n = 2). The median interval between the date of intravenous iron isomaltoside infusion and the date of surgery was 23 (15–31) days. The median dose of iron isomaltoside given to patients was 1000 (1000–1000) mg.

There were no observed differences between the two groups (Table 1). Of the 40 patients, 34 (85%) patients had low ferritin level < 30 μg.l^-1^ and 6 (15%) patients had suboptimal iron stores (ferritin 30–100 μg.l^-1^, transferrin saturation < 20%). Among patients with low iron stores, 4 had evidence of inflammation (CRP > 5 mg.l^-1^). The median waiting time for surgery was 27 (18–34) days in the treatment group and 23 (17–28) days in the control group (*P* = 0.341). There were no reported adverse events during the study period for iron therapy and blood transfusions.

**Table 1:** Characteristics and preoperative data of patients receiving iron isomaltosides or usual care.

	Iron therapy (n = 20)	Usual care (n = 20)
Mean (SD) age; years	68.4 (6.8)	69.8 (12.6)
Sex; male (n, %)	15 (75%)	9 (45%)
Mean (SD) weight; kg	60.4 (11.2)	55.7 (12.9)
Mean (SD) height; cm	161.6 (8.8)	157.4 (7.4)
Mean (SD) body mass index; kg.m^-2^	23.1 (3.8)	22.4 (4.6)
ASA physical status (n, %)		
1	3 (15%)	1 (5%)
2	11 (55%)	13 (65%)
3	6 (30%)	6 (30%)
Comorbidites (n, %)		
Hypertension	11 (55%)	11 (55%)
Diabetes mellitus	9 (45%)	7 (35%)
Ischaemic heart disease	2 (10%)	1 (5%)
Previous stroke	1 (5%)	1 (5%)
Diagnosis (n, %)		
Non-rectosigmoid tumour	6 (30%)	7 (35%)
Rectosigmoid tumour	11 (55%)	12 (60%)
Synchronous tumour	3 (15%)	1 (5%)
Stage IV disease (n, %)	0 (0%)	2 (%)
Tumour T stage (n, %)		
I	1 (5%)	4 (20%)
2	1 (5%)	1 (5%)
3	14 (70%)	10 (50%)
4	4 (20%)	5 (25%)
Tumour N stage (n, %)		
0	10 (50%)	11 (55%)
1	8 (40%)	5 (25%)
2	2 (10%)	4 (20%)
Median (IQR) tumour size (longest); cm	4.5 (3.0–6.8)	3.5 (3.0–4.0)
Mean (SD) baseline haemoglobin; g.l^-1^	108 (14)	105 (14)
Median (IQR) baseline ferritin; μg.l^-1^	20.9 (12.6–27.5)	11.6 (6.6–19.7)
Median (IQR) baseline transferrin saturation; %	9.5 (5.0–12.8)	10.0 (6.3–15.0)
Baseline C-reactive protein > 5 (n, %)	7 (37%)	4 (24%)

**Fig 2:**
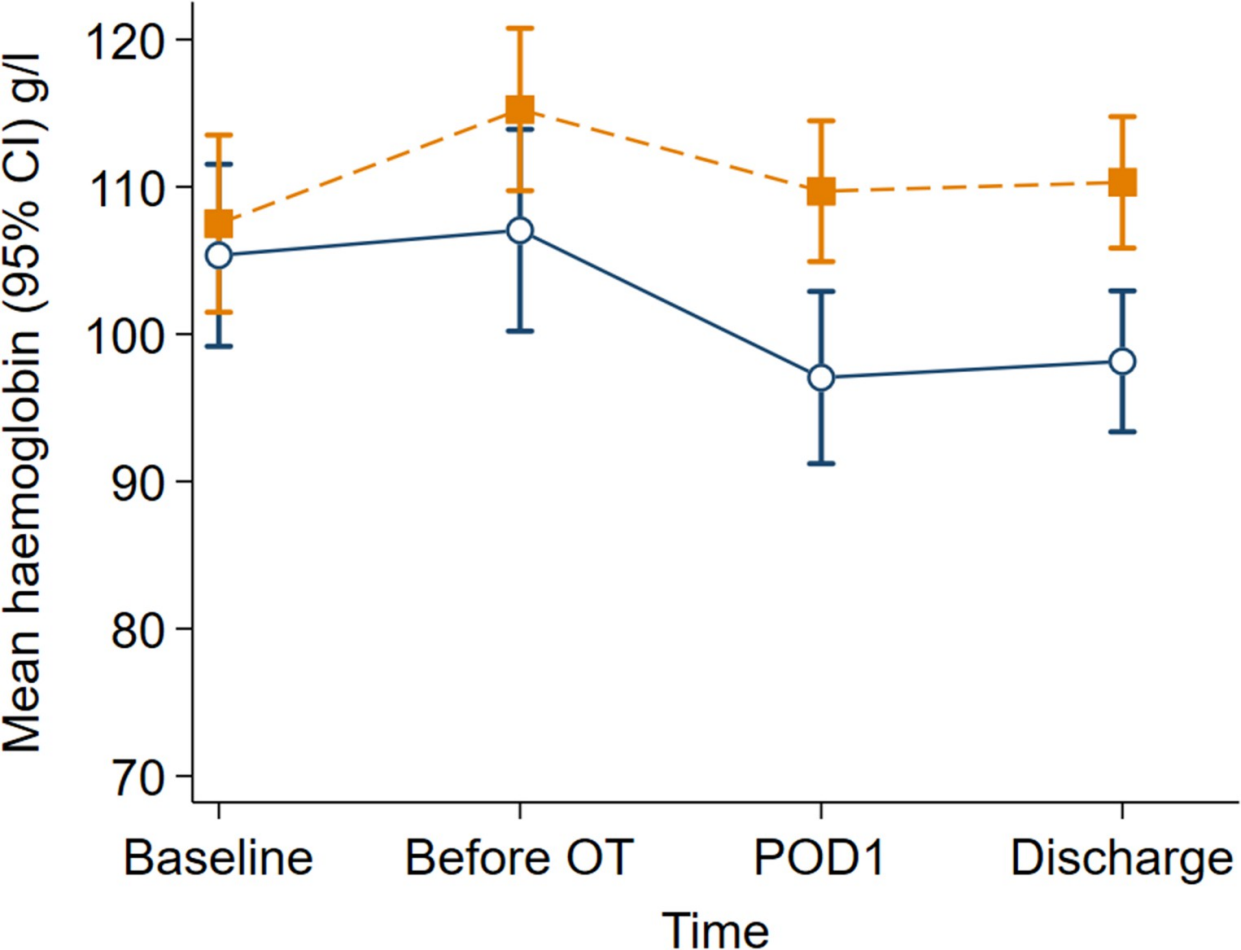
Haemoglobin concentrations (g.^-1^) over time by treatment groups. Data are mean with error bars showing the 95% confidence interval. Intravenous iron isomaltoside (filled orange squares with dash line); control (unfilled navy circles with solid line). OT, operating theatre; POD1, postoperative day one.

**Fig 3:**
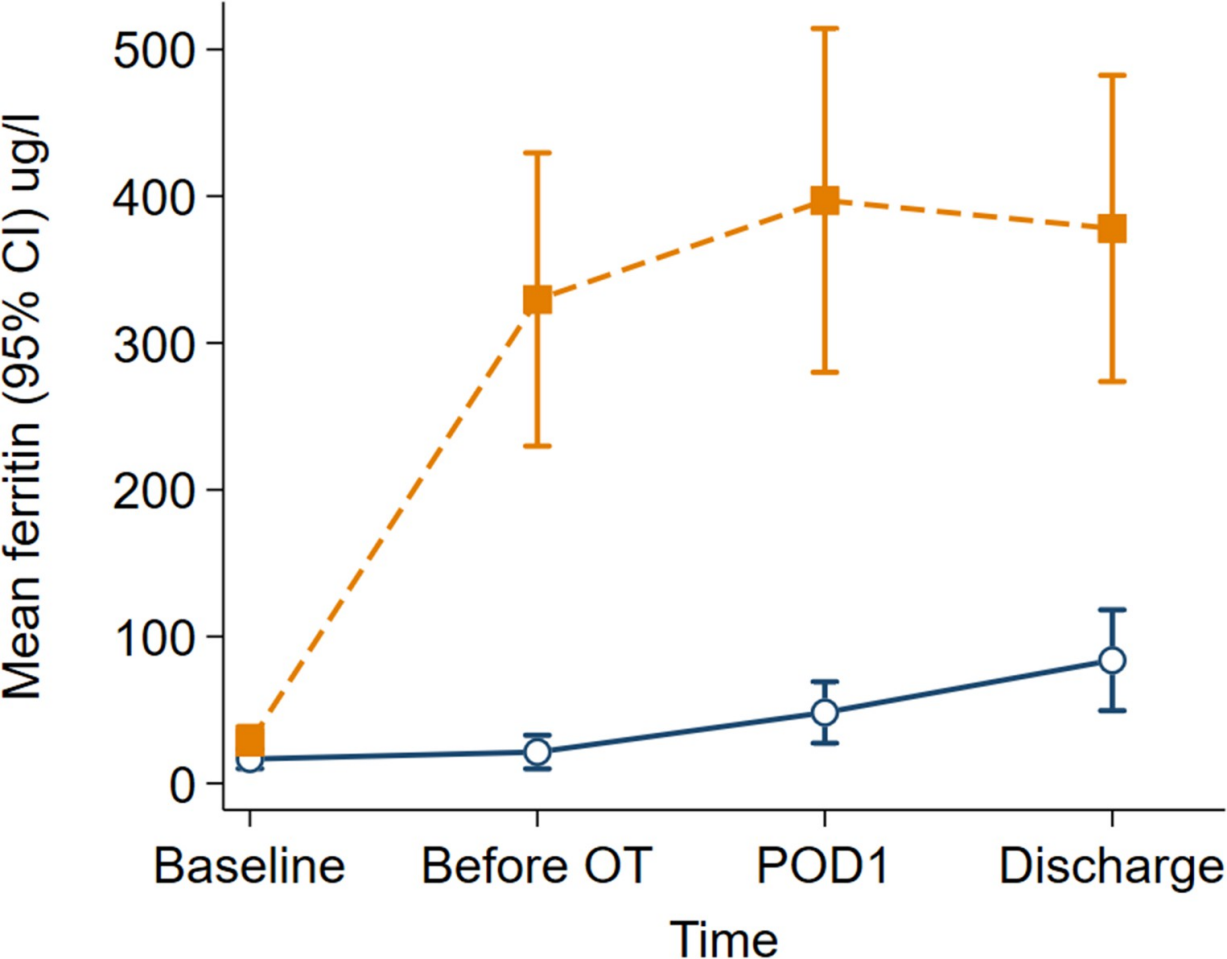
Ferritin concentrations (μg.^-1^) over time by treatment groups. Data are mean with error bars showing the 95% confidence interval. Intravenous iron isomaltoside (filled orange squares with dash line); control (unfilled navy circles with solid line). OT, operating theatre; POD1, postoperative day one.

The haemoglobin and ferritin concentrations were higher in the iron isomaltoside group than the control group across all perioperative time points (group*time interaction *P* = 0.042 and *P* < 0.001, respectively). The mean haemoglobin change from baseline to before surgery was higher in the iron isomaltoside group (7.8, 95% CI: 3.2 to 12.3 g.l^-1^) than the control group (1.7, 95% CI: -1.9 to 5.3 g.l^-1^) [mean difference 6.1, 95% CI: 0.3 to 11.8 g.l^-1^; *P* = 0.040; Fig 2]. The ferritin change from baseline to before surgery between groups was large in favour of the iron isomaltoside group (mean difference 296.9, 95% CI: 200.6 to 393.2 μg.l^-1^; *P* < 0.001; Fig 3]. There were significant perioperative changes in transferrin saturation over time between the treatment and control groups (group*time interaction *P* < 0.001). The overall mean difference in transferrin saturation concentration between the iron isomaltoside and control groups was 4.8% (95% CI: 1.8% to 7.8%).

The number of patients who needed packed red blood cells transfusions during the perioperative period was 2 (10%) in the iron isomaltoside group and 6 (30%) in the control group (*p* = 0.235). The proportion of patients requiring packed red blood cell transfusions in each group at various times is described in Table 2. The total number of units transfused over the perioperative period in the iron isomaltoside and control groups were 3 and 9, respectively. One patient in the treatment group was admitted to the intensive care unit for aspiration pneumonia, sepsis and ileus management (surgical complication graded 3 in Table 2). The proportion of poor recovery (QoR-15 <118) was similar between the treatment (13/20) and control (11/20) groups (*P* = 0.748).

**Table 2:** Intraoperative and postoperative data in patients receiving iron isomaltoside or usual care.

	Iron therapy (n = 20)	Usual care (n = 20)	*P* value
Operation; laparoscopic	14 (70%)	18 (90%)	0.235
Median (IQR) duration of operation; min	189 (170–261)	173 (149–255)	0.327
Median (IQR) blood loss; ml	20 (10–200)	18 (10–38)	0.444
Red blood cell transfusions			
Preoperative	1 (5%)	3 (15%)	0.605
Intraoperative	0 (0%)	0 (0%)	N/A
Postoperative	1 (5%)	4 (20%)	0.342
Surgical complications			
Infection	6 (30%)	4 (20%)	0.465
Ileus	4 (20%)	3 (15%)	1.000
Any	11 (55%)	8 (40%)	0.342
Surgical complication grade			
0	9	12	
1	7	6	0.636
2	3	2	
3	1	0	
Median (IQR) QoR-15 score (0–150)	107 (100–124)	115 (103–125)	0.547
Median (IQR) postoperative length of stay; days	10 (5–19)	7 (5–10)	0.289
DAH30; days	20 (10–25)	23 (20–25)	0.461
Hospital readmission within 30 days	1 (5%)	1 (5%)	1.000

DAH30, Days (alive and) at home within 30 days of surgery; QoR-15, 15-item Quality of Recovery

## Discussion

This pilot trial demonstrated the safety and effect of using intravenous iron isomaltoside therapy for IDA in patients with colorectal cancer despite the slow recruitment rate over the study period. Data completeness was high. We found significant changes in perioperative haemoglobin, ferritin and transferrin saturation concentrations in favour of iron isomaltoside. There were no significant differences between groups in the clinical and patient-centred outcomes as this pilot trial was not powered to detect a difference in postoperative outcomes. No iron therapy- or blood transfusion-related adverse events were reported.

The recruitment process of our pilot trial study protocol requires improvement. The recruitment rate was slower than expected despite 414 elective colorectal cancer surgeries being performed over the study period. In our pilot trial, case screening took place after confirmation of the surgical plan to avoid drop-outs. However, a small proportion of patients presented with significant tumour complications (bleeding and obstruction requiring early operation) who were excluded from the study during screening as the waiting time to operation was less than 3 weeks. To maximize the recruitment rate and minimize possible selection bias in a future study, the logistics of recruiting patients will require further inter-department communication improvements during the early screening process.

The inclusion criteria of at least 3 weeks waiting time to operation was designed to allow time for iron replenishment and haematopoiesis. Seven (35%) patients received iron therapy within 3 weeks of surgery as their operations were bought forward unexpectedly, which may account for the conservative rise in preoperative haemoglobin. A previous study suggested that haemoglobin response after intravenous iron isomaltoside at 1, 2, 3, 4 and 8 weeks was approximately 20%, 50%, 70%, 85% and 100% respectively [20]. Nevertheless, the preoperative haemoglobin change of 6.1 (95% CI: 0.3 to 11.8) g.l^-1^ is comparable to a positive finding comparing ferric carboxymaltose to usual care [10] or placebo [21]. In contrast, intravenous iron sucrose therapy was unlikely to change haemoglobin concentration in a RCT subgroup of anaemic patients [9].

Although all our patients had IDA, a small proportion had anaemia of chronic inflammation with iron deficiency. A post-hoc analysis of the amount of blood loss (ml) showed that patients having anaemia of chronic inflammation with iron deficiency had higher median blood loss (115, IQR 30–313) than patients with true IDA (15, IQR 10–50; *P* = 0.031). A recent small observational study of patients undergoing colorectal surgery suggested that intravenous iron therapy was least effective in patients with ‘probable anaemia of inflammation’ in comparison with patients with ‘probable functional iron deficiency’ or with true IDA in order of magnitude of increasing haemoglobin concentration [22]. In the presence of malignancy, upregulation of hepcidin reduces dietary iron absorption and trapping of iron store [23]. Whether there is a need to further refine treatment strategies for patients having anaemia of chronic inflammation with iron deficiency, such as using recombinant erythropoietin, requires further study in this study population.

There is increasing attention to perioperative iron deficiency without anaemia, which may be a disease in its own right [8]. In non-anaemic patients undergoing colorectal resection, the prevalence of iron deficiency was high (74%) [24]. The baseline, postoperative Day 3 and discharge haemoglobin concentrations were found to be lower in the iron deficient group than in the iron replete group (*P* = 0.01, *P* = 0.05 and *P* = 0.01, respectively) in an observational study [24]. In another pilot RCT (n = 60) of iron isomaltoside given one day or on the day of elective cardiac surgery, smaller decline in haemoglobin concentrations during the first month after surgery in the isomaltoside group compared with the placebo group (*P* = 0.01) was shown [25]. In patients without anaemia but demonstrating iron deficiency, it is plausible that some degree of anaemia may occur during the perioperative period on the basis of decreasing haematopoiesis. This may potentially expose them to reduced exercise capacity and an increased risk of adverse outcomes after surgery. However, there is currently a paucity of prospective observational data supporting this hypothesis. The NATO trial (ACTRN12618001997246) is an observational trial underway to determine the risk of poor postoperative outcome in non-anaemic iron deficient colorectal cancer patients [26].

During the conduct of our current pilot trial, there were two publications of larger scale multi-centred RCTs. The PREVENTT trial (n = 487) on preoperative intravenous iron to treat anaemia before major abdominal surgery [21] reported a minimal haemoglobin concentration response to the intravenous iron carboxymaltose compared to the placebo (mean difference 4.7, 95% CI: 2.7 to 6.8 g.l^-1^), which was similar to our pilot trial findings. There were no differences in the risk of mortality or blood transfusion (co-primary endpoint), postoperative complications, DAH30, health-related quality of life and safety outcomes [21]. There were fewer readmissions at 8 weeks in the treatment group, which the author commented requiring further investigation. The previously published IVICA trial [11] reported a follow-up study comparing quality of life data between groups, with an increase in quality of life scores found in patients treated with preoperative ferric carboxymaltose compared to those receiving oral ferrous sulphate therapy [27].

Given this new evidence emerging during the planning and conduct of our pilot trial, there are several issues that need to be addressed when designing future research studies on perioperative iron deficiency management. First, we restricted the inclusion criteria to patients with IDA and excluded patients who received previous oral iron therapy for clinical homogeneity in our pilot trial but this limited recruitment rate and generalisability of the results. Further research is required to explore the transition between non-anaemic to anaemic states between pre-admission screening and surgery in this surgical population. Current research is underway to examine the association between non-anaemic iron deficiency in colorectal cancer patients and postoperative outcomes [26]. If an association exists, extending the eligibility criteria to non-anaemic iron deficiency patients could be considered to increase the recruitment rate and applicability of the results. Second, given the relatively variable time interval from diagnosis to operation, and potential beneficial effect of intravenous iron on longer term recovery phase up to 2 to 6 months shown by PREVENTT trial [21], it may be worthwhile studying the effect of intravenous iron therapy given at different time points during the perioperative period (e.g. more than 3 weeks preoperatively, less than 3 weeks preoperatively and within 1 week after surgery). Third, the combination of iron therapy with recombinant erythropoietin may maximize treatment effect on haemoglobin rise and recovery, especially in patients suffering from chronic inflammation with colorectal malignancy. Fourth, given the current evidence that perioperative intravenous iron improves haemoglobin level and potentially quality of life and recovery, without a demonstrable difference in transfusion rate, a more meaningful primary endpoint of future study would be a patient-centred outcome such as health-related quality of life (EQ-5D) [28] and World Health Organization Disability Schedule (WHODAS) version 2.0 [29] instead of haemoglobin rise and transfusion rate. Finally, to capture any late complications and hospital readmissions, we need to consider collecting other study end points including days (alive and) at home within 90 days after surgery (DAH90) [24, 28, 30], and other quality of life scores up to 6 months after surgery.

This pilot RCT demonstrated the safety and effect of iron isomaltoside on perioperative changes in haemoglobin, ferritin and transferrin saturation concentrations. Given the long recruitment and study process of this pilot trial, and new evidence and interest on the topic emerging during the study process, we conclude that our current pilot study trial protocol is less feasible. To better address the clinical interest, future clinical trial examining iron isomaltoside as a perioperative intervention on patient-centred outcomes in colorectal cancer patients may be feasible with the suggested changes to trial protocol.

## Supporting information

S1 Checklist CONSORT checklist. CONSORT pilot and feasibility trials checklist.
(PDF)

S1 File (DOCX)
