# Supplementary material for: Perioperative changes in haemoglobin and ferritin concentrations from preoperative intravenous iron isomaltoside for iron deficiency anaemia in patients with colorectal cancer: A pilot randomised controlled trial
Source: PLoS One. 2022 Jun 30;17(6):e0270640. doi: 10.1371/journal.pone.0270640 (PMC9246107; doi:10.1371/journal.pone.0270640)
Supplement: S1 File — (DOCX) [file pone.0270640.s002.docx]

**Submission Date/version: 10^th^ February, 2018/Version 1**

**Study Title:** Efficacy of preoperative intravenous iron isomaltoside in colorectal cancer surgical patients with iron deficiency anaemia compared to standard care: a pilot randomized controlled trial

**Principal Investigator:** Dr. Pui Lam Polly FUNG (1)

**Co-investigators:** Prof. Anna LEE (2), Dr. Vivian Nga Man LAU (1), Dr. Wing Wa LEUNG (3), Prof. Wing Chung Tony MAK (3)

(1) Department of Anaesthesia and Intensive Care, Prince of Wales Hospital, Hong Kong

(2) Department of Anaesthesia and Intensive Care, The Chinese University of Hong Kong

(3) Division of colorectal surgery, Department of Surgery, The Chinese University of Hong Kong

**Area of Research:** Perioperative medicine - patient blood management

**Abstract:** Iron deficiency anaemia is a common condition among colorectal surgical patient. Untreated anaemia would lead to increase in blood transfusion, surgical complications and mortality. Treatment with oral iron sulphate is poorly tolerated due to side effects. Intravenous iron supplement provides an alternative way to rapidly replace iron deficit during the preoperative period among surgical patients. Evidence is growing for its effect in rising hemoglobin level and reducing blood transfusion, at the same time supporting its safety profile.

We plan for a single-centered, randomized controlled trial to examine the effect of intravenous iron compared to standard care in terms of hemoglobin level/serum ferritin increment, need for blood transfusion, duration of hospital stay, quality of recovery and surgical complication rate, as well as safety profile among colorectal cancer surgical patients in Hong Kong. We propose the following pilot RCT for exploring the effect size and study process in conducting the above-mentioned large-scale RCT.

**1. Background**

Iron deficiency is the most common nutritional deficiency in the world, with approximately two billion people worldwide suffering from the condition according to World Health Organization data in 2001.^1^

The human body contains about 3500mg of iron (50mg/kg body weight); two-thirds of which exists as part of hemoglobin, 10% are stored in myoglobin and various enzymes involved in cellular respiration, while the remaining 20% are store as ferritin and haemosiderin inside liver, bone marrow and macrophages in the reticuloendothelial system.^2, 3^ Ferritin is a predominantly intracellular protein that stores iron and releases it in a controlled fashion. It acts as a buffer against iron overload and iron deficiency in humans. Small amounts of ferritin are also secreted into the plasma acting as iron carriers. The serum ferritin level is positively correlated with the total body iron store. A low serum ferritin level is thus a useful marker of reflecting iron deficiency.

One-third of patients undergoing non-cardiac surgery suffers from anaemia, of which iron deficiency or sequestration were the main contributors (75%).^4^ Among this population, the colorectal surgical patients are especially prone to iron deficiency due to an increased iron loss from tumour bleeding, and a reduced iron intake due to poor dietary intake or gastro-intestinal malabsorption. Erythropoiesis is disrupted as a result, leading to iron deficiency anaemia, typically manifested with microcytic hypochromic red cells and low serum ferritin level.

Oxygen carrying capacity in blood is decreased in anaemic patients, which leads to decreased oxygen delivery to tissues. This may have detrimental effects on surgical outcomes. Anaemic patients are more prone to develop cardiac event, and it is associated with an increased risk of 30-day mortality.^5^ Although allogenic blood transfusion can readily correct perioperative anaemia, it was associated with an increased risk of cancer-related mortality (OR 1.71, 95% CI: 1.43-2.05), postoperative infections (OR 3.27, 95% CI: 2.05-5.20), surgical re-intervention (OR 4.08, 95% CI: 2.18-7.62), and all-cause mortality (OR 1.72, 95% CI: 1.55-1.91) in a systematic review of 20,795 colorectal cancer patients.^6^ Thus, preoperative optimization of anaemia is a key concept in the patient blood management strategy to minimize perioperative blood transfusions and its related complications.

Timely correction of preoperative iron deficiency is a possible intervention with the aim to boost up haemoglobin level before surgery. The conventional management of iron deficiency is oral iron supplementation. However, oral ferrous sulphate is often not well tolerated by patients due to its gastrointestinal side effects including constipation and dyspepsia. Thus iron replacement via oral route is often ineffective due to low adherence, and variable absorption. Moreover, replacement via the oral route is slow and may not be adequate to replace iron deficit within short time frame of intervention before operation.

Intravenous iron therapy is an alternative, which allows more efficient iron replacement as a higher dose of iron can be supplemented in a single intravenous dose, and bypasses gastrointestinal absorption. Free iron could not be given via intravenous route, as it would lead to acute toxicity. Therefore, parenteral formulae contain iron molecules embedded in carbohydrate matrix structure, which allows iron to be released in a gradual manner. Iron isomaltoside 1000(Monofer^®^) is an unbranched oligosaccharide with mean molecular weight of 1,000Da, with iron strongly bound within the molecules. Compared to other formulae in which the iron are more loosely bound, iron isomaltoside potentially causes less oxidative stress and toxicity, allowing higher dose to be given over a short period of time. For example, iron isomaltoside can be given at a dose of 20mg/kg body weight, which is higher than that of ferric carboxymaltose, which is 15mg/kg body weight.^7^ Most published studies of perioperative use of intravenous iron therapy showed a positive effect in increasing the hemoglobin level and reducing the number of blood transfusions.^8^

Among colorectal cancer patients, several randomized controlled trials and cohort studies showed favourable results supporting the use of preoperative intravenous iron therapy. Keeler et al.’s conducted a RCT (n=116) showing a higher haemoglobin rise in IV iron group (1.55g/dL, IQR 0.93-2.58) than the oral iron group (0.5g/dL, IQR -0.13-1.33) among colorectal cancer surgical patients.^9^ A prospective, multicenter, observational study (n=266) in patients undergoing elective colon cancer surgery showed a significant reduction in transfusion rates (9.9% vs 38.7%) and length of stay (8.4 vs 10.9 days) in the IV ferric carboxymaltose group compared to oral iron.^10^ A recent retrospective analysis published by Laso-Morales et al in 2017^11^ showed that anaemic colorectal cancer patients who received IV iron were able to achieve similar haemoglobin level as non-anaemic patients on day of surgery and remained similar at postoperative day 30.

In Hong Kong, there has been a rising trend in the number of patients diagnosed with colorectal cancer over the past decade. The number of colorectal cancer new case per year has risen from 3,706 in 2005 to 5,036 in 2015, such that colorectal cancer is now the top cancer in Hong Kong. ^12^ At Prince of Wales Hospital, the majority of elective resection surgeries for colorectal cancer are performed with minimally invasive approach, including laparoscopic surgery or robotic assisted laparoscopic surgery. Due to technological advances in surgical instruments, the laparoscopic approach has become the new standard of care instead of the open approach even for low-lying tumours. Blood loss is much reduced and blood transfusion rate is much lower compared to the value quoted in most studies published overseas (>30%). Early recovery after surgery (ERAS) is implemented in most cases, such that the length of hospital stay is short. The above characteristics makes the currently published evidence, which has a higher baseline blood transfusion rate, longer length of stay, and a higher rate of open surgeries, less applicable to our patient population.

**2. Study Objectives**

This pilot study aims to explore the feasibility of conducting a large scale randomized controlled trial to determine the efficacy and safety of intravenous iron isomaltoside(Monofer®) therapy for the colorectal cancer surgical patients with iron deficiency anaemia compared to standard care.

As discussed above, the patient population in Prince of Wales Hospital is characterized by a majority of laparoscopic surgery with low blood loss and transfusion rate, as well as short hospital stay at baseline. A smaller effect size in terms of reduction in transfusion rate and hospital stay is thus expected, which implies that a large sample size would be required to conduct a RCT with adequate statistical power. Before embarking on such a large-scale research project, it is necessary to conduct a feasibility trial to estimate recruitment rate, sample size and allow proper logistics management.

There are two areas to be assessed in this pilot trial including study process and the predicted effect size.

2.1. Study process:

The number of patients diagnosed with colorectal cancer listed for elective resection per month will be studied, and the prevalence of iron deficiency anaemia among these patients will be recorded. All of the eligible patients satisfying the recruitment criteria stated below would be invited to join the pilot study, and the refusal rate will be noted. The information is required to calculate the recruitment rate of the study. The median waiting time for surgery will also be calculated. We envision that a recruitment rate of more than 60%, 5 patients per month and a median waiting time of 4 weeks will be necessary for conducting a larger RCT.

2.2. Effect size:

In this pilot trial, we plan to explore the effect size of the primary outcomes for the proposed RCT, including haemoglobin rise and serum ferritin rise associated with the intravenous iron treatment group compared to standard care. The secondary outcomes to be measured will include perioperative blood transfusion rate, hospital length of stay, quality of recovery scores, surgical complications, adverse reactions and days (alive and) at home within 30 days of surgery (DAH_30_). As stated before, the baseline transfusion rate for elective colorectal surgeries in our population is lower compared to international studies, therefore it would be inaccurate to use existing data to estimate the sample size.

**3. Study Design**

3.1. Methodology

A single-centered, superiority, 2-arm, randomized controlled trial is planned to determine the efficacy and safety of preoperative intravenous iron isomaltoside (Monofer®) therapy compared to standard care (control) in colorectal cancer patients undergoing elective tumour resection surgery. The study design is outlined in the diagram in Figure 1.

Figure 1. Overview of study design

Other parameters measured: surgical complication, Days (alive and) at home within 30 days of surgery (DAH30)

For the pilot study to assess the feasibility of conducting the actual large-scale trial, 40 patients (20 in the treatment arm and 20 in the control arm) will be recruited. The study design of the pilot study would be identical to the above-proposed trial with the aim to explore the study process and the predicted effect size.

3.2. Study population

Consecutive patients diagnosed with colorectal cancer listed for elective curative tumour resection surgery (including open and laparoscopic approach) will be screened for eligibility based on the criteria listed below. Baseline blood tests (including: complete blood counts, renal and liver function, iron profile (serum ferritin, transferrin saturation/TSAT), serum B12 and folate) will be collected as a routine preoperative workup.

3.3. Inclusion criteria (All criteria must be fulfilled)

- Age >18 years old with written informed consent
- Anaemia defined as: hemoglobin concentration < 13g/dL (same cut-off for both male and female patients according to the International consensus statement on the perioperative management of anaemia and iron deﬁciency)^2^
- Laboratory test confirmed iron deficiency: serum ferritin <30mcg/L alone *or* serum ferritin 30-100mcg/L with TSAT < 20%

3.4. Exclusion criteria (None of the criteria must be fulfilled)

- Pregnancy or lactation
- Other known causes of anaemia apart from iron deficiency: untreated B12/folate deficiency, hemolytic disease, hemoglobinopathy/thalassemia, chronic renal failure on dialysis
- Presence of iron overload (serum ferritin > 300g/dL or TSAT > 50%); known hemochromatosis
- Previous or ongoing iron replacement/use of erythropoietin within 12 weeks before recruitment
- Known hypersensitivity towards iron isomaltoside
- Significant liver function derangement (AST/ALP exceeding three times upper limit of normal range)
- Use of preoperative immunosuppressant therapy
- Participation in another ongoing interventional clinical trial(s)
- Patients with less than 2 weeks waiting time to surgery

3.5. Consent

Eligible subjects will be recruited as early as possible when all the above criteria have been fulfilled. They will be approached by research personnel for obtaining written informed consent. Information sheet with detailed explanation of the objectives, duration, data collections, intervention involved and safety issues will be provided for the recruited patients. They will be provided with contact information of the research team such that they can ask questions, or withdraw from the trial if needed.

3.6. Definition of primary and secondary outcomes:

*3.6.1. Effect size*

Primary Outcomes:

- Preoperative change in hemoglobin concentration (g/dL) and change in serum ferritin (mcg/L), which is, the difference between the value at diagnosis and preoperative day.

Secondary Outcomes:

- Units of red blood cells transfused in perioperative period in the two groups, defined as:

1. Preoperative - from randomization to operation;

2. Intraoperative – inside operating theatre and recovery room;

3. Postoperative - from arrival to ward after operation to discharge.

- Duration of hospital stay (days).
- Quality of recovery as measured by questionnaire (QoR-15(Chinese)) completed on postoperative period. The QoR-15 (See Appendix A) includes the items measuring pain, physical comfort, physical independence, psychological support and emotional state.^13^ The QoR-15 score ranges from 0 to 150 and takes about 3 minutes to complete.^13^ The validity (convergent, construct, discriminant), reliability (internal consistency, split-half, test-retest), responsiveness, acceptability and feasibility properties have been well established.^13^
- Incidence of adverse reactions/serious adverse events to intravenous iron administration and adverse reaction to blood transfusions (Adverse reactions: any unintended responses related to drug/blood products administrations e.g. skin rash, pain, neurological/respiratory/cardiovascular symptoms. Serious adverse events: life-threatening/require additional period of hospitalization/results in disability/mortality eg. Anaphylaxis, acute hemolytic transfusion reactions, organ failure etc.).
- Rate of surgical complications recorded and graded according to Clavien Classification of Surgical Complications.^14^ (See Appendix B)
- Days (alive and) at home within 30 days of surgery (DAH_30_)
- A parameter incorporating the duration of initial hospital stay associated with the index surgery, re-hospitalization due to post-discharge complications, discharge to institutional care and early deaths within 30 days post operatively. It has been validated as an effective patient-centered outcome measure for perioperative trials. ^15^
- Method of calculation – refer to Appendix C

*3.6.2. Study process:*

- Patient recruitment rate (number of recruited patient/total number of eligible patients x 100%)
- Number of patients recruited per month.
- Median waiting time to surgery (days).

3.7. Randomization, allocation concealment and blinding

Recruited patients would be prospectively randomized to either preoperative intravenous iron isomaltoside(treatment group) or standard care(control group). The 1:1 allocation will be performed by one of the investigators, not involved in patient care or study assessment, using a computer program PASS 14.0 (NCSS, Kaysville, UT). The treatment allocation will be concealed in sealed opaque envelopes and handed over to third party anaesthetists (other than the investigators) for arranging pre-operative intravenous iron therapy for patients in the treatment arm, or no therapy in the control arm.

Records related to the iron administration will be kept away from the investigators and the parent surgical team for the duration of the study period to reduce performance bias. Patients in both arms will not be blinded to the treatment they may receive in the management of perioperative anaemia, be it intravenous iron in the treatment group, or any necessary perioperative blood transfusions in either groups. An alert in the patient’s electronic records will be implemented to remind the parent surgical team and the case anaesthetist that the patient is participating the study, however the both parties will be blinded to the patients’ group assignment, to avoid interference to their transfusion decision.

Unblinding will be done only in the condition where clinician believes that clinical management depends importantly on whether the patient received intravenous ferric isomaltoside. Unblinding will be achieved by contacting the investigators, and any unblinding event and the reason of unblinding will be recorded.

3.8. Baseline patient demographics and clinical data collection

After patient recruitment, baseline characteristics including age, sex, body weight and height, ASA grading, important past health comorbidities (hypertension, diabetes mellitus, significant cardiovascular diseases, known hemolytic conditions/hemoglobinopathy), medication history will be collected. The information regarding baseline hematological profile (hemoglobin level, ferritin level) at the time of recruitment will be recorded.

3.9. Standard perioperative care apart from the study interventions

Standard anaesthetic preoperative assessment will be carried out either in the preoperative assessment clinic or by the list anaesthetist on the day before surgery, according to the routine practice in our hospital for all elective colorectal cases. Patients are allowed to continue their usual medications as directed by the parent surgical team and case anaesthetists. Upon recruitment, patients will not receive iron supplements other than intravenous iron isomaltoside.

Following the existing practice, patients for elective colorectal cancer resection operations are admitted to surgical wards one day before the operation. The second batch of study blood tests (including complete blood count, iron profile) will be taken along with cross match on admission. Bowel preparation will be undertaken if deemed necessary by the parent team.

General anaesthesia will be provided by anaesthetists blinded to the study allocation, without restrictions on the anaesthetic agents and analgesic modalities to be used. As per usual practice within the department, intravenous induction will be achieved with propofol and fentanyl, followed by maintenance with sevoflurane/desflurane. Muscle relaxation with non-depolarizing muscle relaxant (atracurium/cisatracurium/ rocuronium) will be maintained throughout the operation, followed by reversal with neostigmine/atropine at the end of operation. Analgesia will be achieved by multimodal approach, by a combination short and long acting opioids (fentanyl/morphine), tramadol, paracetamol, non-steroidal anti-inflammatory drugs (NSAIDs) and local anaesthetics (intravenous infusion and/or local infiltration) at the discretion of the list anaesthetists. Antiemetics will be given as routine either with ondansetron, dexamethasone or metoclopramide.

After the surgery, patients will be managed in surgical wards under the colorectal team. Fluid sips and diet will be resumed and stepped up as soon as tolerated usually starting on postoperative day one. Study blood sample (hemoglobin level, iron profile) will be taken with the other routine blood tests on postoperative day one. Patient will be discharged home as long as adequate recovery is achieved and discharge criteria are met as decided by the parent surgical team. Before patient discharge, patients will be approached by research personnel to fill in a questionnaire (QoR 15 Chinese version) assessing the quality of recovery outcome on third day after surgery.

Throughout the perioperative period, the decision to transfuse blood is based on clinical judgment by the parent surgical team and list anaesthetists, who are blinded to the patients' group allocation. There is no specific transfusion trigger defined in this study protocol as it might vary among patients with different needs, for example, patient with history of ischemic heart disease or stroke may have a higher transfusion trigger.

3.10. Intervention: intravenous iron isomaltoside(Monofer^®^)

Patients randomized to the treatment group will be given a single dose of intravenous iron isomaltose in the time frame of 2-10 weeks before operation date. The dose will be determined by the patient’s body weight: > 50kg: 1000mg; <50kg: 20mg/kg body weight, to be infused over 30 minutes. The infusion would take place in the day surgical unit and patients will be closely monitored. Vital signs would be charted before, at 15 minutes during infusion and immediately after the infusion. Patients will be discharged home on the same day if no adverse reactions are observed.

3.11. Control: Standard care

Patient randomized to the control arm will follow the standard perioperative care as described above and the perioperative management they received will be identical to the treatment arm except no intravenous iron isomaltoside will be given.

3.12. Follow up/assessment

Data on the primary and secondary outcomes as listed above would be collected. Investigators will access the patients’ records via the Hospital Authority computer system and the hardcopy documents in the wards. Data on the hematological profile throughout study period (on diagnosis, preoperative day, post-operation day one, on discharge) will be retrieved from the computer records. The number of red cell transfusion will be retrieved from the computer records as well as counterchecking with anaesthetic records/progress notes. The length of hospital stay (for the operation and DAH_30_) will be counted by referring to computer patient records.

The quality of recovery is assessed by a self-reported quality of recovery questionnaire (QoR-15)^13^, which assess different dimensions of recovery including pain, physical comfort, physical independence and mental well-being.

**4. Statistical analysis**

Missing data will be checked and imputed to preserve power. The Shapiro-Wilk test will be used to check data for normality. Generalized estimating equations will be used to examine the changes in haemoglobin level and serum ferritin levels over time. Expected skewed data, such as the quality of recovery score, the number of days of hospital stay, units of transfusion and DAH_30_, will be reported as median and interquartile range, and compared between treatment arms by Mann-Whitney U tests. Two-tailed χ^2^ test will be used to analyze categorical data between groups. All of the analysis would be intention-to-treat and with a statistical significance level defined at P<0.05.

**5. Expected impact on study/patient care**

This pilot study is crucial for refining the design for a larger scale, prospective RCT in the future. The effect size in the primary outcome measures obtained in in this study will help determine sample size for the larger study; based on sample size and the recruitment rate, the enrolment duration can be estimated. Although intravenous iron has been advocated as a more convenient and effective alternative to oral iron therapy in terms of raising ferritin level and preoperative haemoglobin level, it remains to be determined whether these increases in laboratory parameters translates into significant improvement in patient outcomes or quality of recovery. Intravenous iron does come with additional cost and admission. Whether this can be offset by a reduction in transfusion rate, and hospital length of stay will require a further cost-effectiveness analysis in future. The study may provide useful evidence in writing a clinical protocol for managing preoperative iron deficiency anaemia in colorectal cancer surgical patients with the ultimate aim of reducing exposure to allogenic blood products and improvement in patient outcomes.

**6. Potential hazards, Safety and audit**

6.1. Safety profile of iron isomaltoside (Monofer ^®^)

Among the six currently available formulations of intravenous iron available, serious adverse events are very rare (38 per 10^6^ administrations). About 1:200 patients receiving intravenous iron experience complement activation related pseudo-allergy which manifests as arthralgia, myalgia and flushing, which should not be confused with true anaphylaxis. These symptoms typically subside without interventions.^2^ There are concerns on the effects of oxidative stress caused by intravenous iron but there is no evidence of clinical adverse event resulting from it. Some suggested that intravenous iron may promote infection by providing iron to pathogenic bacteria^16^, however there is no increase in infection observed in a meta-analysis of 103 trials published from 1965 to 2013 (including 19,253 patients).^17^

6.2. Audit

The investigators will permit trial-related monitoring, audits, CREC/IRB review and regulatory inspections, providing direct access to source data/documents.

**7. Confidentiality and records**

Raw electronic data will be encrypted and access is only provided to investigators to ensure confidentiality. The investigators are responsible for archiving the trial documents. The raw data and trial documents will be retained for at least 3 years for retrieval of data. Only group data will be published.

Only the following people will have access to the raw data or information:

1. Investigators and the team members

2. The Institutional Review Board

3. Subjects have the rights of access to personal data and known study results, if and when needed.

**8. Ethics**

Written informed consent will be obtained from the patient. Patients may withdraw from the project without prejudice. We seek approval from the The Joint Chinese University of Hong Kong – New Territories East Cluster Clinical Research Ethics Committee and the trial will be conducted in compliance with the Hospital Authority Guide on Research Ethics, the Declaration of Helsinki, and the International Conference on Harmonisation – Good Clinical Practice (ICH-GCP) Guidelines.

**9. References**

1. World Health Organization. *Iron Deficiency Anaemia: Assessment, Prevention, and Control. A Guide for Programme Managers.* Geneva: WHO, 2001.

2. Muñoz M, Acheson AG, Auerbach M, Besser M, Habler O, Kehlet H, Liumbruno GM, Lasocki S, Meybohm P, Rao Baikady R, Richards T, Shander A, So-Osman C, Spahn DR, Klein AA. International consensus statement on the peri-operative management of anaemia and iron deficiency. Anaesthesia. 2017;72:233-247.

3. Kam P, Powers I. Chapter 9. Principles of Physiology for the Anaesthetist. 3^rd^ Ed. CRC Press, 2015.

4. Garcia-Erce JA, Laso-Morales MJ, Gomez-Ramrez S, Nunez- Matas MJ, Munoz M. Analysis of the prevalence and causes of low preoperative haemoglobin levels in a large multicentre cohort of patients undergoing major non-cardiac surgery. Transfusion Medicine 2016; 26(Suppl. 1): 48.

5. Gupta PK, Sundaram A, Mactaggart JN, Johanning JM, Gupta H, Fang X, Forse RA, Balters M, Longo GM, Sugimoto JT, Lynch TG, Pipinos II. Preoperative anemia is an independent predictor of postoperative mortality and adverse cardiac events in elderly patients undergoing elective vascular operations. Ann Surg. 2013;258:1096–1102.

6. Acheson AG, Brookes MJ, Spahn DR. Effects of allogeneic red blood cell transfusions on clinical outcomes in patients undergoing colorectal cancer surgery: a systematic review and meta-analysis. Ann Surg. 2012;256:235 – 244.

7. Kalra PA, Bhandari S. Efficacy and safety of iron isomaltoside (Monofer(®)) in the management of patients with iron deficiency anemia. Int J Nephrol Renovasc Dis. 2016 ;9:53-64.

8. Peters F, Ellermann I, Steinbicker AU. Intravenous iron for treatment of anemia in the 3 perisurgical phases: a review and analysis of the current literature. Anesth Analg. 2017 Dec 19. doi: 10.1213/ANE.0000000000002591. [Epub ahead of print].

9. Keeler BD, Simpson JA, Ng O, Padmanabhan H, Brookes MJ, Acheson AG; IVICA Trial Group. Randomized clinical trial of preoperative oral *versus* intravenous iron in anaemic patients with colorectal cancer. Br J Surg 2017;104: 214–221.

10. Calleja JL, Delgado S, del Val A, Hervás A, Larraona JL, Terán Á, Cucala M, Mearin F; Colon Cancer Study Group. Ferric carboxymaltose reduces transfusions and hospital stay in patients with colon cancer and anemia. Int J Colorectal Dis. 2016;31:543–551.

11. Laso-Morales M, Jericó C, Gómez-Ramírez S, Castellví J, Viso L, Roig-Martínez I, Pontes C, Muñoz M. Preoperative management of colorectal cancer-induced iron defciency anemia in clinical practice: data from a large observational cohort. Transfusion. 2017;57:3040-3048.

12. Hong Kong Cancer Registry, Hospital Authority, Hong Kong

13. Stark PA, Myles PS, Burke JA. Development and psychometric evaluation of a postoperative quality of recovery score: the QoR-15. Anesthesiology. 2013;118:1332-40.

14. Dindo D, Demartines N, Clavien PA. Classification of surgical complications: a new proposal with evaluation in a cohort of 6336 patients and results of a survey. Ann Surg 2004; 240: 205-13.

15. Myles PS, Shulman MA, Heritier S, et al. Validation of days at home as an outcome measure after surgery: a prospective cohort study in Australia. BMJ Open 2017;7:e015828. doi: 10.1136/bmjopen-2017-015828

16. Litton, E., Xiao, J., and Ho, K.M. Safety and efficacy of intravenous iron therapy in reducing requirement for allogeneic blood transfusion: systematic review and meta-analysis of randomised clinical trials. BMJ. 2013; 347: f4822

17. Avni T, Bieber A, Grossman A, Green H, Leibovici L, Gafter-Gvili A. The safety of intravenous iron preparations: systematic review and meta-analysis. Mayo Clin Proc. 2015;90:12-23.

**10. Appendices**

**Appendix A – Quality of recovery (QoR-15) Patient Survey**

| *Part A: How have you been feeling in the last 24 hours?*  (0 to 10, where: 0 = none of the time [poor] and 10 = all of the time [excellent]) |
| --- |
| 1. Able to breathe easily  2. Been able to enjoy food  3. Feeling rested  4. Have had a good sleep  5. Able to look after personal toilet and hygiene unaided  6. Able to communicate with family or friends  7. Getting support from hospital doctors and nurses  8. Able to return to work or usual home activities  9. Feeling comfortable and in control  10. Having a feeling of general well-being |
| *Part B: Have you had any of the following in the last 24 hours?*  (10 to 0, where: 10 = none of the time [excellent] and 0 = all of the time [poor]) |
| 11. Moderate pain  12. Severe pain  13. Nausea or vomiting  14. Feeling worried or anxious  15. Feeling sad or depressed |

Stark PA, Myles PS, Burke JA. Development and psychometric evaluation of a postoperative quality of recovery score: the QoR-15. Anesthesiology. 2013;118:1332-40.

**Appendix B – Clavien Classification of Surgical Complications**

| Grade I | Any deviation from the normal postoperative course without the need for pharmacological treatment or surgical, endoscopic, and radiological interventions.  (allowed therapeutic regimens: antiemetics, antipyretics, analgesics, diuretics, electrolytes, physiotherapy, wound infection opened at bedside) | |
| --- | --- | --- |
| Grade II | Requiring pharmacological treatment with drug other than such allowed for Grade I complications. E.g blood transfusion, TPN | |
| Grade III | Requiring surgical, endoscopic or radiological intervention. | |
|  | Grade IIIa | Intervention not under general anaesthesia. |
|  | Grade IIIb | Intervention under general anaesthesia. |
| Grade IV | Life-threatening complication requiring ICU management | |
|  | Grade IVa | Single organ dysfunction. |
|  | Grade IVb | Multi-organ dysfunction. |
| Grade V | Death of a patient. | |
| Suffix “d” | If the patient suffers from a complication at the time of discharge | |

Dindo D, Demartines N, Clavien PA. Classification of surgical complications: a new proposal with evaluation in a cohort of 6336 patients and results of a survey. Ann Surg 2004; 240: 205-13.

**Appendix C – Method of calculation of the ”Days at Home within 30 days of Surgery” (DAH_30_)**

*DAH_30_ is calculated by incorporating days of hospitalization and mortality from the date of the index surgery (= Day 0). The calculation method is as follow:*

1. If a patient dies within 30 days of surgery, irrespective of how many days they have spent at home postoperatively, DAH_30_ should be scored as zero (0).

2. Otherwise DAH_30_ is calculated by the number of days a patient spent at home post-discharge from the index surgery (counting from day of index surgery).

Example 1

Patient died on day 2 after their surgery whilst still an inpatient, DAH_30_ would be 0.

Example 2

Patient was discharged home on Day 6 after index surgery but was subsequently readmitted for 4 days for surgical complications, DAH_30_ would be 20 (i.e. 30-6-4=20).

Example 3

If a patient has complications and spends 16 days in hospital, and then is transferred to a nursing facility for rehabilitation, and spend 24 days there before finally being discharged home, they would be assigned 0 DAH_30_. (30-16-24 = -10, but the minimum value of DAH_30_ should be 0).

Example 4

Patient had planned re-admission (eg. removal of a stent or secondary closure of a fistula) within 30 days of surgery should have these days subtracted from the total DAH_30_. If a patient is discharged from hospital on Day 13, and is electively re-admitted two weeks later (Day 27) for a further 2 days, they would be assigned 15 DAH_30_ (i.e. 30-13-2=15).

Myles PS, Shulman MA, Heritier S, et al. Validation of days at home as an outcome measure after surgery: a prospective cohort study in Australia. BMJ Open 2017;7:e015828. doi: 10.1136/bmjopen-2017-015828
